# Supplementary figures and images for: Kin recognition: Neurogenomic response to mate choice and sib mating avoidance in a parasitic wasp
Source: PLoS One. 2020 Oct 26;15(10):e0241128. doi: 10.1371/journal.pone.0241128 (PMC7588116; doi:10.1371/journal.pone.0241128)

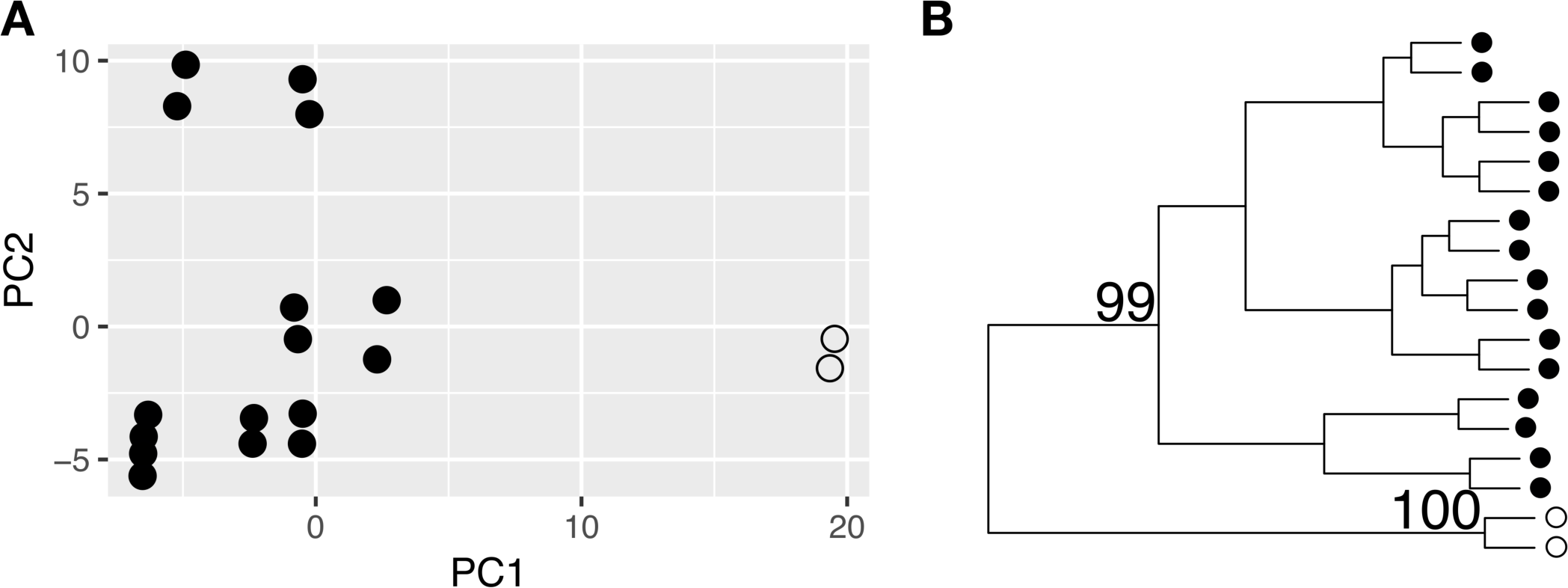

Supplement: S1 Fig — Multivariate analysis based on the expression profiles of the 500 genes with the highest variance across all samples showed that one biological replicate corresponding to females courted by related males (2 empty circles representing 2 technical replicates) is far from the other points (full circles) A) in the plan defined by the two first axes of the principal component analysis and B) in the sample hierarchical clustering dendrogram. Significant statistical support of outlier status is indicated by an approximately unbiased p-value with one million multiscale bootstrap replicates. (TIF) [file pone.0241128.s001.tif]

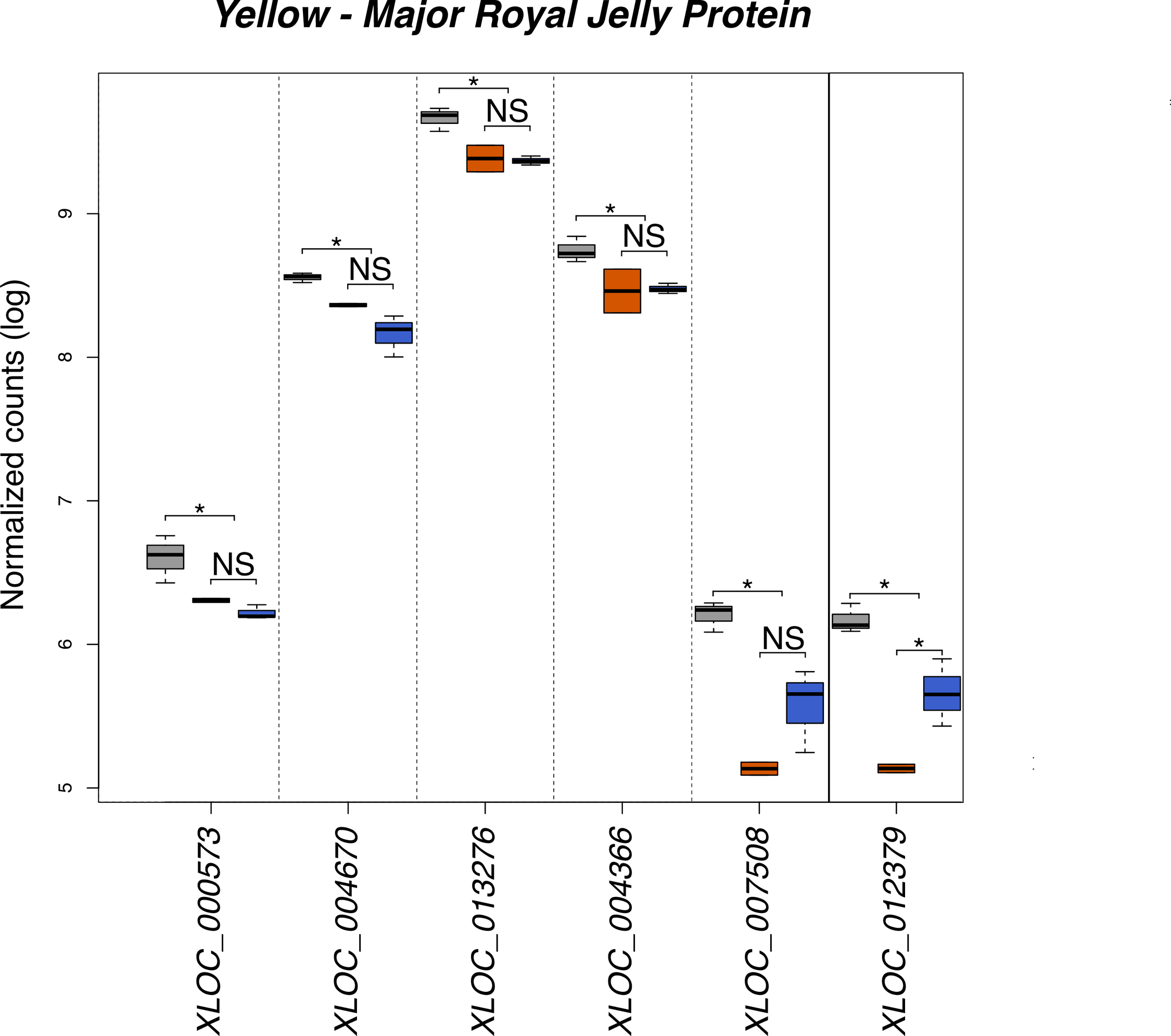

Supplement: S2 Fig — Boxplot colours indicate biological condition: grey, isolated females; blue, females courted by unrelated males; orange, females courted by related males. The Y axes show the normalized counts after log transformation and VST normalization, and the boxplot whiskers show the range of reads between biological replicates. *, P-adj (FDR) < 0.01; NS, not significant. (TIF) [file pone.0241128.s002.tif]

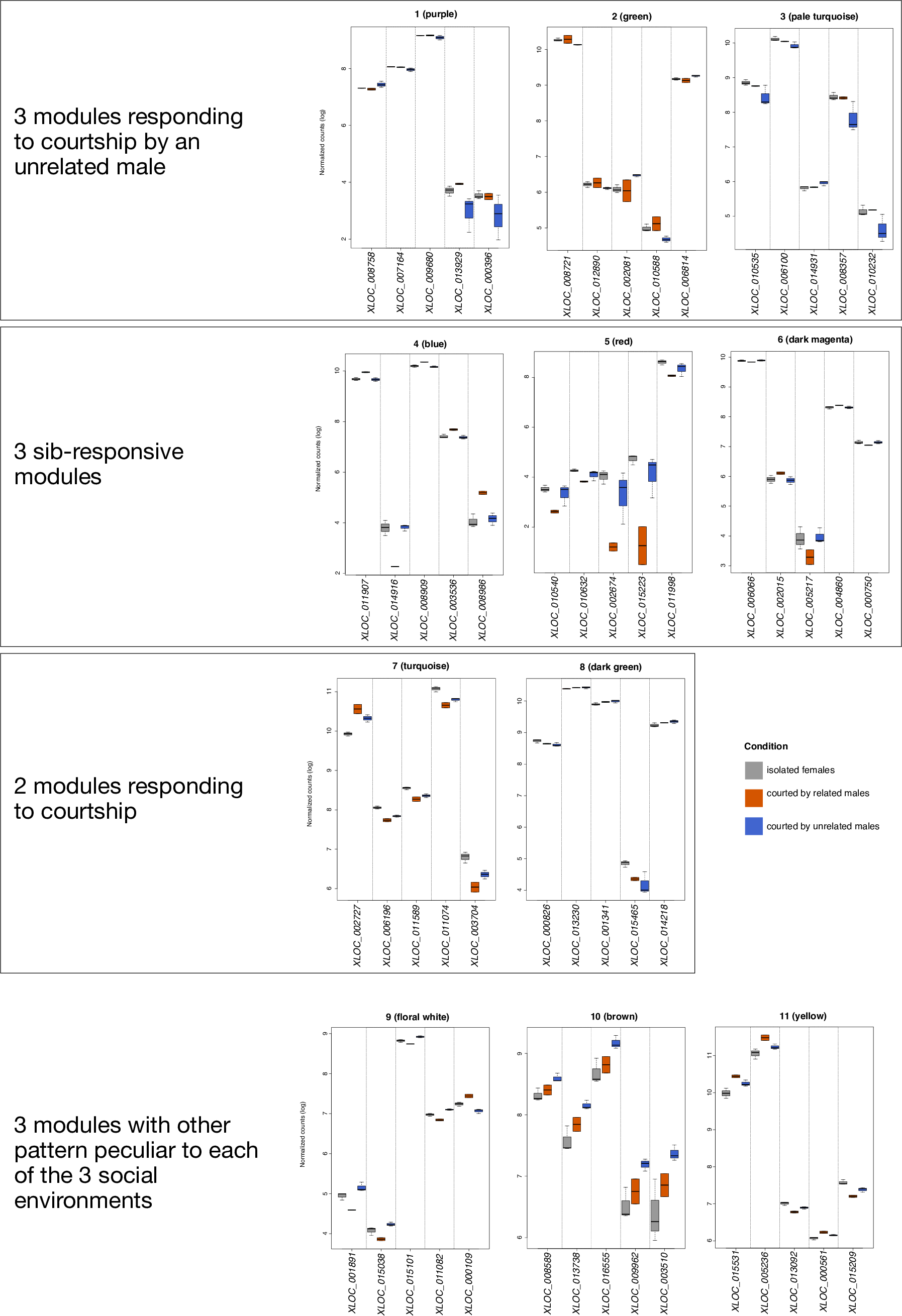

Supplement: S3 Fig — The Y axes show the normalized counts after log transformation and VST normalization, and the boxplot whiskers show the range of reads between biological replicates. (TIF) [file pone.0241128.s003.tif]
