# Supplementary material for: Kin recognition: Neurogenomic response to mate choice and sib mating avoidance in a parasitic wasp
Source: PLoS One. 2020 Oct 26;15(10):e0241128. doi: 10.1371/journal.pone.0241128 (PMC7588116; doi:10.1371/journal.pone.0241128)
Supplement: Attachment — Submitted filename: Response_to_reviewers.docx [file pone.0241128.s013.docx]

*Dear William J. Etges,*

*We want to thank the reviewers as well as the editor for their corrections. We hope that this revised version includes all the changes requested.*

*A comprehensive response to the specific items provided by the 2 reviewers has been addressed. We have responded to each item in details in the following section (part 6).*

*On behalf of the authors,*

*Aurore Gallot, PhD.*

PONE-D-20-01602R1
Kin recognition: neurogenomic response to mate choice and sib mating avoidance in a parasitic wasp
PLOS ONE

Dear Dr. gallot,

Thank you for submitting your manuscript to PLOS ONE.  We invite you to submit a revised version of the manuscript that addresses the points raised during the review process.

I agree with both external referees that the authors have done a solid job of responding to the reviewers' comments and criticisms. Both reviewers have suggested a few more issues that need addressing before a final decision can be made.  I think the MS is much clearer and the results are better described for your readers.

Please submit your revised manuscript by Oct 22 2020 11:59PM. If you will need more time than this to complete your revisions, please reply to this message or contact the journal office at [plosone@plos.org](mailto:plosone@plos.org)<<mailto:plosone@plos.org>>. When you're ready to submit your revision, log on to <https://www.editorialmanager.com/pone/> and select the 'Submissions Needing Revision' folder to locate your manuscript file.

Please include the following items when submitting your revised manuscript:

 *   A rebuttal letter that responds to each point raised by the academic editor and reviewer(s). You should upload this letter as a separate file labeled 'Response to Reviewers'.
 *   A marked-up copy of your manuscript that highlights changes made to the original version. You should upload this as a separate file labeled 'Revised Manuscript with Track Changes'.
 *   An unmarked version of your revised paper without tracked changes. You should upload this as a separate file labeled 'Manuscript'.


If you would like to make changes to your financial disclosure, please include your updated statement in your cover letter. Guidelines for resubmitting your figure files are available below the reviewer comments at the end of this letter.

If applicable, we recommend that you deposit your laboratory protocols in [protocols.io](http://protocols.io)<<http://protocols.io/>> to enhance the reproducibility of your results. [Protocols.io](http://Protocols.io)<<http://protocols.io/>> assigns your protocol its own identifier (DOI) so that it can be cited independently in the future. For instructions see: <http://journals.plos.org/plosone/s/submission-guidelines#loc-laboratory-protocols>

We look forward to receiving your revised manuscript.

Kind regards,

William J. Etges
Academic Editor
PLOS ONE

Reviewers' comments:

Reviewer's Responses to Questions


Comments to the Author

1. If the authors have adequately addressed your comments raised in a previous round of review and you feel that this manuscript is now acceptable for publication, you may indicate that here to bypass the “Comments to the Author” section, enter your conflict of interest statement in the “Confidential to Editor” section, and submit your "Accept" recommendation.

Reviewer #1: All comments have been addressed

Reviewer #2: (No Response)

________________________________


2. Is the manuscript technically sound, and do the data support the conclusions?

The manuscript must describe a technically sound piece of scientific research with data that supports the conclusions. Experiments must have been conducted rigorously, with appropriate controls, replication, and sample sizes. The conclusions must be drawn appropriately based on the data presented.


Reviewer #1: Yes

Reviewer #2: Yes

________________________________


3. Has the statistical analysis been performed appropriately and rigorously?


Reviewer #1: Yes

Reviewer #2: Yes

________________________________


4. Have the authors made all data underlying the findings in their manuscript fully available?

The PLOS Data policy<<http://www.plosone.org/static/policies.action#sharing>> requires authors to make all data underlying the findings described in their manuscript fully available without restriction, with rare exception (please refer to the Data Availability Statement in the manuscript PDF file). The data should be provided as part of the manuscript or its supporting information, or deposited to a public repository. For example, in addition to summary statistics, the data points behind means, medians and variance measures should be available. If there are restrictions on publicly sharing data—e.g. participant privacy or use of data from a third party—those must be specified.


Reviewer #1: Yes

Reviewer #2: Yes

________________________________


5. Is the manuscript presented in an intelligible fashion and written in standard English?

PLOS ONE does not copyedit accepted manuscripts, so the language in submitted articles must be clear, correct, and unambiguous. Any typographical or grammatical errors should be corrected at revision, so please note any specific errors here.


Reviewer #1: Yes

Reviewer #2: Yes

________________________________

6. Review Comments to the Author

Please use the space provided to explain your answers to the questions above. You may also include additional comments for the author, including concerns about dual publication, research ethics, or publication ethics. (Please upload your review as an attachment if it exceeds 20,000 characters)

Reviewer #1: The authors have responded to all my comments in a satisfactory manner.

There is one exception with regards to the sample that was removed. The text should mention why the sample represents a problem during preparation, and not due to biological variation, given that both technical replicates are very similar, and both are outliers.

*We explicitly added in the manuscript why the outlier sample is problematic and has been removed (l. 248-249).*

Minor comments

All the minor comments has been taken in account in the new version of the manuscript

Line 259:did not applied -> did not apply

*We have corrected (l. 271).*

Line 289: this should not be the full transcriptome, but the part of the transcriptome that was included in the analysis. The authors mention that they corrected for this, so I suspect this is text that has not been updated.

*We have corrected (l. 302).*

Lines 356-358: Please mention in the text the direction of high expression for the genes that are discussed.

*The direction of high expression is now described for all the discussed genes. We added this information for the genes ebony, DAT, 5-HT1A and serotonin receptor 1A* *(l. 372-375).*

Line 425: Showed -> shows, illustrated -> illustrates, denoted -> denotes, indicated -> indicates

*We have corrected (l. 444-447).*

Line 480: Veltos -> Veltsos

*We have corrected (l. 505).*

Fig 3A: Please check the top left outlier gene. Is it reliable? If not consider removing it to shorten the y axis.

*We controlled the outlier gene but we do not considered that it is aberrant given that the corresponding sequence encode and open reading frame that perfectly match to the mitochondrial gene CO1. Considering that the gene annotation is reliable, we did not remove the outlier point, and did not rescale the figure either.*

Fig 4: the symbols and numbers overap, please use smaller text.

*We stretched the y axis and used a smaller text in the figure 4b.*

Reviewer #2: This is a much improved version of a previously reviewed manuscript: “Kin recognition: neurogenomic response to mate choice and sib mating avoidance in a parasitic wasp”. I have a few remaining minor comments that the authors should address.

Minor points:

Line 241: “All pairs of technical replicates were merged before….” Not “has been merged”.

*We have corrected (l. 252).*

Line 249: missing the words “of all” between expression and transcripts. It should read: “tested for differential expression of all transcripts with an average level of expression above 10 reads per library”

*We have corrected (l. 261).*

Figure 1 legend: Please state which color represents which treatment in the figure legend instead of stating that treatments are represented by different colors.

*We added which color represent which treatment in the figure legend (l. 350-351).*

Line 381: This section is a little unclear. Are all 22 of these GO terms only associated with 2 genes? Or are they associated with some of the other 479 differentially expressed genes as well?

*Only the 2 GO ‘Reproductive Behaviour’ and ‘Male Mating Behaviour’ are associated with 2 genes. We clarified this section (l. 401).*

Lines 549-550: The authors need to mention the decades of research on the molecular pathways involved in kin-recognition in Hydractinia symbiolongicarpus and Botryllus schosseri. This topic has been well studied in colonial marine invertebrates since at least 1994 (Mokady & Buss, 1994; Fagan & Weissman, 1997; Cadavid et al., 2004; Litman, 2006; Nicotra et al., 2009; Nydam et al., 2017, to name a few of the many papers on this topic).

*We mentioned research on kin recognition in colonial invertebrates in the introduction (l. 47-51).*

It looks like the authors included both the original 4 figures and the revised figures, but failed to mention which figures were the old figures and which figures were the new figures. It looks like the second set of figures are the new figures. Assuming that is true, the color scheme is definitely more color-blind friendly, though Figure 4b is still a little difficult to read. I suggest stretching the y-axis so the cluster numbers are easier to match to bars.

*We stretched the y axis and used a smaller text in the figure 4b.*

The new S1 figure is useful, as is S3. I appreciate their addition.
